# Living with primary brain calcification with *PDGFB* variants: A qualitative study

**DOI:** 10.1371/journal.pone.0275227

**Published:** 2022-10-07

**Authors:** Tomiko Takeuchi, Hisami Aoyagi, Yoshimi Kuwako, Isao Hozumi

**Affiliations:** 1 Faculty of Nursing, Toyama Prefecture University, Toyama City, Toyama, Japan; 2 Laboratory of Medical Therapeutics and Molecular Therapeutics, Gifu Pharmaceutical University, Gifu, Japan; 3 Department of Neurology, Graduate School of Medicine, Gifu University, Gifu, Japan; Dokkyo Medical University: Dokkyo Ika Daigaku, JAPAN

## Abstract

**Introduction:**

Primary brain calcification (PBC) is a rare and intractable neurodegenerative disease. *SLC20A2* and *PDGFB* are two major causative genes. As there is no effective treatment to avoid further progression or to prevent the onset of the disease, the patients may experience psychological distress. There is a qualitative study on the experiences of patients with primary brain calcification with *SLC20A2* variants. However, the experiences of patients with *PDGFB* variants of the disease have not been explored. The purpose of this study is to identify the experiences of patients with *PDGFB* variants after diagnosis.

**Materials and methods:**

Semi-structured interviews were conducted once or twice a year for three years with five patients over the age of 21. The data were analyzed using inductive qualitative methods.

**Results:**

Seven categories, 15 subcategories, and 129 codes were extracted. The seven categories are as follows: [Shock at hearing the term ‘brain calcification’ for the first time], [Anxiety regarding the risk of heredity], [Anxiety, along with severe headaches, and various other symptoms], [Gratitude for the family members who care], [Accepting the disease as a non-life-threatening illness], [Feeling alienated due to the rare intractable disease], and [Modifying lifestyle due to the illness].

**Discussion:**

The most stressful aspect of the disease was the headache that persisted even with the use of analgesics, which was different from patients with the *SLC20A2* variants. In addition, we found unique concepts such as anxiety regarding the risk of heredity and a feeling of alienation due to the rare and intractable disease.

## Introduction

Primary brain calcification (PBC) is also known as Fahr’s disease, idiopathic basal ganglia calcification (IBGC), and primary familial brain calcification (PFBC). PBC is a rare neurological neurodegenerative disease. It is characterized by brain calcification in the basal ganglia, cerebellar dentate nuclei, and thalami, among others. Some of the patients, especially those with familial PBC, are found to have causative genes. Some PBC patients are asymptomatic, but others experience symptoms such as parkinsonism, cerebellar symptoms, cognitive impairment, psychosis, seizures, or chronic headaches. PBC is defined with the differential diagnoses of parathyroid disorders, mitochondrial disorders, infectious diseases, inherited metabolic syndromes, physiological calcification, and others [1–3].

The causative genes of PBC have been successively identified over the past decade; namely, *SLC20A2* [4], *PDGF (Platelet-derived growth factor) RB* [5], *PDGFB* [6], *XPR1* [7], *MYORG* [8], *JAM2* [9]. *SLC20A2*, *PDGFRB*, *PDGFB*, and *XPR1* are the autosomal dominant causative genes, while *MYORG* and *JAM2* are the autosomal recessive genes. Around the world, *SLC20A2* accounts for 40%, *PDGFB* accounts for 10%, and these two genes account for half of the total patients with *PFBC* or familial *IBGC* (FIBGC) [2, 10, 11].

At present, there is no effective therapeutic agent for preventing the onset and suppressing the progression of calcification in the brain, so the psychology of the patients who are given this diagnosis is complicated. How do patients, who are told that they have a genetic disease, perceive the current situation in which there is no cure? We reported the experience of living with the disease of patients with *SLC20A2* variants in 2016 [12]; however, no similar successive studies have been reported since then.

Respecting the patient’s experience and listening with sympathy is the important key to effective treatment and care for patients with intractable diseases who have not found a curative treatment. The intensive qualitative research on "Illness narratives" (Narrative Based Medicine; NBM) is required in order to understand the experience of patients living with rare intractable diseases, how they face these illnesses, and find supportive methods and effective mental treatments. Concomitantly, we should recognize that NBM and EBM (Evidence-Based Medicine) complement each other in medical treatment and nursing care.

In this study, we qualitatively analyze continuous semi-structured interview data obtained from patients with *PDGFB* variants and clarify the characteristics and commonalities of each category. It is very important for us as a general social resource to share the experience of living with various symptoms and the thoughts of patients who are suffering from a rare, intractable, and familial disease. In addition, it is significant to analyze the psychological and social characteristics among patients with different gene variants in the same disease category.

## Methods

### Aims

The aim of this study was to clarify the psychological and social characteristics of patients with primary brain calcification who have *PDGFB* pathological variants. In addition, we attempted to reveal, in particular, the experience of living with various symptoms.

### Design

The qualitative study design that describes and summarizes phenomena as they are in daily language was used to clarify experiences of people living with a rare intractable disease [13, 14].

### Participants and recruitment

Five patients over the age of 20 participated in the study. Each provided written, informed consent. Four of the patients were referred to a specialist, and one looked up information on the internet to see a specialist. Prior to the study beginning, *PDGFB* variants had been already discovered by the genetic study with the approval of the Ethics Committee and with written consent from the participants [11, 15].

### Data collection

After introducing herself, one researcher, who has taken interview training, made a request for verbal and written informed consent. After obtaining consent, she conducted semi-structured interviews. At this time, she also received consent from each patient for her to obtain medical information from doctors. She secured a quiet place where privacy could be kept and recorded the interviews on an IC recorder with permission. While emphasizing the freedom to talk about experiences of the illness, she guided them to talk about their thoughts regarding: "how the illness was found", "thoughts when they first heard the name of the illness", "current thoughts about the illness", "thoughts for the family after the diagnosis", “what they learned through their experience with the illness”, and “what they hope for medical care and support systems.” The interviews were performed from March 2017 to February 2020. During this period, 3 out of 5 patients were able to undergo two interviews.

### Analysis

Verbatim transcripts were made and qualitative content analysis was performed according to the qualitative method by U. Flick [16]. These verbatim transcripts of the interview, recorded on an IC recorder were done on paper, and the accuracy of the contents was confirmed among the collaborators. After that, (1) the verbatim record was put into MAXQDA10, a qualitative data analysis support software, and the record related to the theme was divided into groups and given names to express the meanings (coding). (2) All the codes were sorted and organized in chronological order. (3) All the codes were compared with each other and summarized by those with similar characteristics. A name indicating the characteristics was given to each group (subcategorization). (4) The passage of time was confirmed for all subcategories, and the subcategories were reassembled in chronological order. (5) Finally, the entire subcategory was reviewed again, compared with each of the other subcategories, summarized by those with similar characteristics, and the names indicating the characteristics were given to the grouped items (categorization).

In order to ensure the quality of the analysis results, we were supervised by collaborators specializing in qualitative research. Moreover, in order to confirm whether the interpretation of the interview data was appropriate, the results were given back to the participants, and a check by researchers was performed [17].

### Ethics approval and consent to participate

The studies were approved by the Ethics Committees of Toyama Prefectural University (approval number: H29-14), Gifu Pharmaceutical University (approval number: 1–25 and 1–26) and Gifu University (approval number: 29–245 and 27–298). The studies were also registered in the University Hospital Medical Information Network (UMIN)-CTR System approved by the International Committee of Medical Journal Editors (ICMJE) (Registered ID; UMIN000022167 and 000030100).”

## Results

### Outline of participants

There were 5 participants in the study: 3 females and 2 males (Table 1).

**Table 1:** Summary of participants with *PDGFB* variants.

ID	1	2^a)^	3	4	5
**Sex**	Female	Female	Female	Male	Male
**Age at detection of calcification**	43	15	19	71	57
**Reason for consultation**	Recommendation from a specialist	Falling	Falling	Bradykinesia	Words don’t come out
**Age of diagnosis**	43	16	38	71	57
**Age of Interview**	47	21	41	76	63
	48	24	43	with his wife	
**Time of Interview (minutes)**	32	40	68	43	32
	34	65	48		
**Symptoms at interview**	Headache, forgetfulness, time sensation disorder, dizziness, tinnitus, perspiration, stumbling, turbulance of letter shape, changes in taste	Headache, pain in the back of eyes, tremor in the left hand, forgetfulness	Headache, forgetfulness, memory decline, stumbling, insomia	Time sensation disorder, forgetfulness, falling down, stumbling, small steps	Words don’t come out, forgetfulness, constipation, stumbling, turbulance of letter shape (at the age of 45, disappearance of headache)
**MMSE**^b)^ **score (years)**	29 (47)	-	30 (41)	23 (71)	28 (57)
	22 (49)		29 (43)		
**Variants**	c.457-1G>T	c.457-1G>T	c.196C>T	c.33_34del	c.160+2 T>A
**Protein**	–	–	p.(Arg66Cys)	p.(Cys12Leufs*19)	–
**PolyPhen-2**	Not applicable	Not applicable	Probably Damaging	Not applicable	Not applicable
**ACMG class**	Pathogenic	Pathogenic	Uncertain significance	Pathogenic	Pathogenic

^a)^ ID2 is ID1’s daughter

^b)^ Abbreviation: MMSE = Mini-Mental State Examination

The clinical manifestation and laboratory data of the participants were described in detail elsewhere [11] except ID3. The summary of the patients is also shown Table 1 and below.

Case ID1 is a mother of five children. ID2 is a daughter of ID1. They both had calcification in their brains and had the same pathological variants (c.457-1G>T). ID1 has suffered from headaches since she was 15 years old and had sometimes taken sedatives. Although no other special symptoms and signs were observed, recently she complained of forgetfulness without cognitive decline in examinations.

ID2 is a daughter of ID1. She visited an emergency unit in a hospital owing to headaches and pain in the back of her eyes that had continued since she was 10 years old. Two years later, she was sometimes unable to walk straight, then would recover. When she (ID2) was found to have *PDGFB* pathogenic variant, her mother (ID1), was also revealed to have the same variant. The data in details in ID1 and ID2 are shown as I-2, and II-2 (Case 2) in family 1, respectively, in the reference [11].

ID3 is a new sporadic case showing a pathological variant (c.196C>T, p.Arg66Cys (Supplementary data 1)). She had a simple head injury at the age of 19 and calcification was identified by a CT scan, according to her medical history. At the age of 38, she visited Gifu University hospital due to severe headaches. Her main complaint was headaches that had continued since her teens. She showed normal physiological examination without mental impairment. Her clinical examination studies showed no specific abnormality except calcification in the brain. ID4 is a sporadic case showing a pathological variant in *PDGFB* gene (c.33_34delCT, p. Cys12 Leufs*19). He had felt unstable going down the stairs for about a year, which led to his consultation. At the time of his first visit, the Mini-Mental State Examination (MMSE) score was 23 points. Mild tremor of his fingers and mild bradykinesia were also observed at the same time. His family has not been examined. His data are shown in detail as Case 3 in sporadic cases in the reference [11]. ID5, like his father, had calcification in the brain. His main complaint was difficulty speaking and forgetfulness. His neurological examination revealed 28 points in MMSE (almost normal mental activity) and mild cerebellar ataxia. His son was also tested owing to a panic disorder since his teens. He was found to have the same *PDGFB* variant as his father. However, as his mother did not want her son to be interviewed, no interview was performed. His data are shown in detail as Case 8 (II-2 in family 1) in the reference [11].

The ages of the participants at the time of the interviews were between 21 and 76 years. It had been 3 to 8 years since they were told that they were suffering from Fahr’s disease or IBGC. Interviews were conducted 1 or 2 times. Interview duration ranged from 32–68 minutes. ID4 was accompanied by his wife who worried about his forgetfulness. She said nothing during the interview.

### Experiences of the patients who were told they suffer from a rare familial disease

As a result of content analysis, 7 categories, 15 subcategories, and 129 codes were extracted. Below, the categories are indicated by [], the subcategories are indicated by << >>, and the narratives are indicated by "".

#### Overview of experiences

Patients were **[shocked at hearing the term ‘brain calcification’ for the first time]**. They were upset by the reality that there was little information about it. A woman (ID3) decided to have an abortion due to **[anxiety regarding the risk of heredity]**. ID1, 2, and 3 experienced **[anxiety, along with severe headaches, and various other symptoms]** due to the consumption of painkillers for severe headaches since their teens. ID1 and 3 always took notes to prevent forgetfulness and had severe anxiety about what would happen if the illness progressed. Patients felt **[gratitude for the family members who care]**. Patients could **[accept the disease as a non-life-threatening illness]** over time. They thought that they had no choice but to ignore it until a curative drug was made. Patients were told they were aging even though there was in fact brain calcification occurring, or their illness was not taken seriously because their disease was not life-threatening. As a result, everyone experienced **[feeling alienated due to the rare intractable disease**]. Some patients also stated they **[modified their lifestyles due to the illness]**, like ID5 who said, “It’s better to not to hide the illness and just be open about it”, and ID2, 3, and 5 who said, “I see the doctor regularly for my family.”

#### Each category

*Category 1*: *[Shock at hearing the term ‘brain calcification’ for the first time]*. Patients took medical examinations due to headaches or fall. They were <<surprised and confused to see calcification images on the CT scan>> (ID2, 3, and 4). On the other hand, ID1, who was advised to take a CT scan by her daughter’s (ID2) doctor, had predicted that she might have calcification. ID5, who had the same symptoms as his deceased father, also predicted that he might have brain calcification.

"I went to a hospital with my dad. There, I was told that I had brain calcification in my head, and the doctor said there was nothing wrong with it, but I was shocked. It was more than 20 years ago. I have been worried about it since then. … It’s true that I was constantly thinking about it. Every time I took a CT scan, I was told that there was something white in my brain”

(ID3).

There are various situations in the process between the notification of calcification in the brain to the definitive diagnosis. Some patients (ID1, 2, 5) were immediately referred to a specialist. ID4 was introduced to a specialist through two hospitals by her doctor. It took more than 20 years for ID3 to find a specialist on the internet. All patients were <<surprised when they first heard the term ‘brain calcification’, and faced the reality that there was little information>> in books or on the internet about it.

*Category 2*: *[Anxiety regarding the risk of heredity]*. A woman who was told by a doctor that she had suffered from familial Fahr’s disease. She had been <<concerned about risks and had anxiety about the heredity of the disease.>> She also had been taking painkillers and tranquillizers due to headaches and mental stress. She decided to have an abortion because of anxiety about the adverse effects of drugs she was taking (ID3). Two patients sometimes fell and complained of headaches (ID2, 3) and were worried that their children might suffer from the same disease.

"My daughter falls quite a bit. She is 2 years old now. She is pretty mischievous and trips and falls pretty frequently. Do you think she may suffer from the disease?"

(ID2).

In addition, all four patients, excluding the 76-year-old man (ID4) with dementia, were told that <<they had a genetic disease but didn’t blame anyone for it.>>

"Now I know that my disease may be from my grandmother who died of Parkinson’s disease… I have no resentment, but I’m very sorry for my daughter who suffers from the same disease”

(ID1).

*Category 3*: *[Anxiety*, *along with severe headaches and various other symptoms]*. Three women said that their <<hardest experience in life was the headaches they’ve had since their teens.>> They had taken over-the-counter painkillers. After consulting with doctors, they were prescribed oral medicines and suppositories for pain. They were able to manage it themselves but continued to suffer from severe temporal headaches (ID1, 2, 3).

"The sound of my heartbeat hurts my temples, you know? It’s a constant pulsing ache. It’s not like it’s going to stop. It always hurts."

(ID3)

All patients, including the 24-year-old woman (ID2), complained of forgetfulness, and was <<always taking notes to coping with forgetfulness>>. An insurance saleswoman (ID1) had trouble with her job due to her forgetfulness at 48 years old and looked for a new job. She got lost when she went to her friend’s house, which she had visited many times in the past (ID1). All of them had difficulties in life due to <<parkinsonism and sensory disturbance>> including hand tremor and falling easily. They were aware of increasing symptoms with age and had <<anxiety about what would happen if it progressed >> (ID1, 2, 3, and 4). Only one patient did not have much anxiety because his father had calcification in the brain as well, but his symptoms were milder than those of his father (ID5).

"There is no medicine to stop the progression. What should I do if I have a seizure? … I sweat from fear. When I read about seizures in books, they said that I may urinate or defecate during a seizure. I’m very scared" (ID1). “I wonder whether I can continue to support myself and my children… and when I start thinking about this, I start to panic. But there’s nothing I can do so I just live with my anxiety"

(ID2).

*Category 4*: *[Gratitude for the family members who care]*. The families of all five patients knew about the illness. ID2 talked to her brothers and sisters who had no symptoms and they accepted it with a positive attitude. Patients expressed <<gratitude toward their families who accepted the illness, and continued to provide care and support to them>> ID4 was grateful to his wife who worried about his dementia and constantly provides care, ID5 was grateful to his mother and wife who accepted the news about his illness matter-of-factly.

"My husband looked up the disease and found research about it on the internet. It showed CT images that were like my pictures. So, my husband said, "We’ll go to the hospital to see a doctor.”, but I said, "The hospital is too far." … But my husband drove me there anyway. We left home at 3 o’clock in the morning and went to the hospital, gently rocked by the car during the drive"

(ID3).

*Category 5*: *[Accepting the disease as a non-life-threatening illness]*. The first interviews were done between 3 and 6 years after receiving a definitive diagnosis from doctors. All the patients took CT scans regularly with their family doctors and were told that calcification had not increased. Based on both the explanations from their family doctors and specialists, who said that "it is not an immediate life-threatening illness" and "a therapeutic medicine is under development", they thought that <<until a curative drug is developed, there is no choice but to try to ignore it>>, taking an evasive coping method (ID1, 2, 3, and 5).

"It doesn’t progress. It doesn’t grow. But we don’t have any medicine and I take painkillers for the headaches. I don’t know if this is a very good way to say it, but I have to pretend it doesn’t exist. I would get an operation if it could be removed. But it is impossible, I know. It’s not like I’ll be bedridden because of it, and I can live pretty normally right now"

(ID3). <<None of the patients wanted to participate in the patient association meetings while they could lead relatively normal lives. >>

"I don’t want to get involved in such meetings. If someone tells me about what’s going to happen or symptoms I’ll experience later on, even if I don’t experience them, I may still dwell on them. I may imagine something wrong. I’d like to ask about suggestions for coping, but if I hear scary things, I may end up dwelling on those"

(ID2).

"If you post new information on the disease on a website, people who want to see it will see it. It’s enough. I have a lot of opportunities to look at things on the internet because I use a personal computer at my company every day. I like to use it"

(ID3).

*Category 6*: *[Feeling alienated due to rare intractable disease]*. One patient had gone to 4 hospitals, but she had been told that <<calcification has no impact on everyday life >> (ID3). When ID5 said at a medical check-up that he suffered from Fahr’s disease, the doctor wasn’t able to fully understand the situation, which left the patient feeling alienated (ID5). ID3 did not talk about the disease to anyone, including friends and school-related people, because she <<never wanted to be known as having a familial intractable disease>> (ID3).

"When I was diagnosed with Fahr’s disease, no hospital treated me. They didn’t even know the name of the disease when I went to a different hospital. I want the public to learn about this disease in the news or something"

(ID1).

"During a medical check-up, a doctor asked me questions. I assumed the doctor really didn’t understand the disease. When I said something about my symptoms, he only said, "Is that so?" He didn’t know about it at all"

(ID5).

*Category 7*: *[Modifying lifestyle due to the illness]*. While some patients did not want people other than their families to know that they had an incurable illness, ID5 felt better telling his boss about the disease. His boss then changed his position to a more suitable one, which allowed him to work until retirement. Because of his experience, he said that <<it was better to talk about the illness, instead of hiding it >> (ID5). In addition, all the patients said that they would <<go for regular medical check-ups for their families. >> He proposed that brain CT should be included in the medical checklist (ID5).

"Calcification was found when I was in junior high school. At that time, I didn’t care about it. I thought I could die without regret. But when I had my second child and ended up with an anxiety disorder, I began to think about various things. Now I go to the hospital to get a CT scan of my head regularly” (ID2).

## Discussion

### Experiences of the patients who were told they suffer from a rare familial disease

Advanced skills are required to diagnose intractable neurological diseases for which there is no cure yet. If not done properly, it can cause psychological damage for patients [18]. Participants in this study with the *PDGFB* variants were told by specialists the name of this disease, which they had never heard of before, except for one patient (ID5). ID5 had lived with his father who had similar symptoms, and explained that the disease had no cure yet. All other patients were confused when they heard the diagnosis. Psychological support is crucial when announcing the name of a disease because patients may feel the need to make unnecessary decisions like one patient who accepted death, and another, who had an abortion in order to prevent the child from having the same fate. After several years, they realized that the disease was not a life-threatening illness because their CT images at the regular check-ups showed the calcified areas were not growing. However, they also experienced symptoms such as continual headaches, progressive forgetfulness, and small steppage gait. Then, it became clear in the study that anxiety about the future continued. One patient (ID1), especially, who was in the child-rearing period, had to work and needed to take notes to combat her forgetfulness and take painkillers so as not to bother others in her workplace. She had a strong desire not to let people in the workplace know about her illness. This led to anxiety, insomnia, and more severe headaches. In addition, in the current situation where there is no cure and the symptoms cannot be alleviated, the following psychological burden is put on the patients: They fear losing their job due to others knowing about the disease, and/or fear of being discriminated against or prejudiced as persons with a familial intractable disease. Moreover, when ID1 saw her child complaining of a fall or headaches, she was worried that her daughter might have inherited it.

A systematic review [19] of FIBGC reported that headaches are more common in *PDGFB* (32.5% of cases), and parkinsonism is more common in *SLC20A2* (21% of cases). The review also found that cognitive impairment is more common in 15% of all cases and depression in 7% of all cases. In the present study, it was repeatedly stated that patients with *PDGFB* variants suffer from headaches such as "pain from heartbeats" and "temple tightening" from a young age, and even analgesics do not relieve these symptoms. In contrast, in six patients with the *SLC20A2* variants that Batla reported in 2017, it was said that "headaches are relieved by oral medication, so it was not necessary to talk to a doctor about them [19]. Very recently, we have also showed that patients with the *PDGFB* variant have a tendency for a higher frequency of headaches [20]. The headaches in PBC may be the secondary headaches caused by the dysfunction of the blood-brain-barrier. However, the frequency in more patients and the mechanism of headaches in PBC should be further clarified in the future.

Taken together, support by a multidisciplinary team, including a genetic counsellor, is necessary from an early stage. Judging from the government collected data at the end of 2018, the ages of patients are widespread, from 30s to late-stage elderly, peaking for those in their 60s [21, 22]. Not only genetic support, but also general counseling and employment support, are important for working patients. When we think about support for the patients, it is necessary for us to take into consideration the results of this study that indicate they do not want to participate in the patient association while they are relatively fine. This result reflects their perception that they may not obtain useful information in the meetings because there is no curative medicine. Moreover, this is because of common beliefs about intractable diseases in our contemporary world. The experience of patients with hereditary diseases and their families is very similar to the experience of patients with chronic diseases and their families. One report says that this experience is related to stigma in society [23]. As such stigma reduces self-esteem and self-efficacy and provokes a sense of social isolation. We must start with efforts to make healthcare professionals understand the disease. For example, we need to build a website that gathers information based on patients’ experience and links to a page within the Japan Intractable Diseases Information Center website (https://www.nanbyou.or.jp/entry/3838, in Japanese) [21]. It is necessary for us to promote awareness-raising activities through disseminating accurate information on the disease. If patients can obtain peer support at home through the website, then social isolation, anxiety, confusion, etc. will improve much more [24].

On the other hand, ID5 spent many years seeing CT images of his father, which showed massive calcification. He also saw his father’s forgetfulness and other symptoms. He felt better about talking to his boss about his illness when it became difficult to speak, and because of this, he could work until retirement age. He said that his father’s symptoms were more severe than his and his father had died of aspiration pneumonia. And yet, he was not so pessimistic about the disease. Therefore, patients can avoid unnecessary anxiety through understanding the actual situation of people who live with the illness. It gives patients a great advantage for understanding how to cope. It would be a beneficial factor for patients to predict the future to some extent and be able to accept it. This can help us inform patients with real, true information. Therefore, support with the above-mentioned multidisciplinary collaboration and information dissemination specific to the disease are required in the patient care.

## Limitations of the research and future issues

At the end of July, 2021, 39 families have been registered in our research as familial idiopathic basal ganglia calcification. In rare intractable diseases, it is difficult for us to find research targets and obtain consent from patients. In the study, we think that we could successfully obtain stories of the valuable experiences of five patients. From the story of patients who had been told that there is no cure for the disease, we find the characteristic concepts of [risks and anxiety about heredity], [anxiety while coping with various symptoms] and [feeling alienated due to the rare intractable disease]. It is a future task to present the chronological changes and elucidate the essence of the new concept through continuous interviews. This might be the most important issue.

## Conclusion

The following characteristics were found in patients with primary brain calcification with *PDGFB* variants.

1. The most painful is the headache that continues even after using analgesics, which differs from patients with the *SLC20A2* gene mutation. In addition to severe headaches, patients had uncontrollable symptoms such as forgetfulness and parkinsonism, and they continued to have anxiety about the future.
2. Since the size of the calcification in the CT image has not changed even after several years, it is not a life-threatening disease. However, patients had a psychological burden regarding the possibility of losing their jobs due to others knowing about the illness, being discriminated against, and experiencing prejudice as a familial intractable disease patient. Also, they were worried that it might be inherited by their children who complain of falls and headaches.
3. Other patients had regular consultations while taking an evasive coping method that did not aggravate their illness, and they strongly hoped that a remedy could be provided for their children.

Based on the above, we believe that support from a multidisciplinary team, including genetic counseling specialists, is needed from an early stage. Genetic support, general counseling, and employment support are also necessary. In addition, it is important to disseminate information about the disease to local residents and medical professionals, other than the specialists, so as to not increase the feeling of alienation due to the rare intractable disease. At this time, considering that some people do not want to participate in the patient association, we will continue to keep in contact with each patient in order to avoid unnecessary anxiety, while also taking into consideration information dissemination on the internet.

## Supporting information

S1 Fig Brain CT scan. This is the Brain CT scan of ID3.
(TIF)

S2 Fig *PDGFB* variant. This is the *PDGFB* Variant of ID3. The search for p. Arg66Cys using PolyPhen-2 indicates probable damage. The variant is classified as a variant of unknown significance in ACMG class. Functional and familial analyses will be dispensable for further studies on *PDGFB* variants.
(TIF)

S1 Table Each category. This is the complete list of research themes and representative quotes.
(TIF)

S2 Table Interview guidelines. This is the entire semi-structured interview.
(PDF)

## Abbreviations

IBGC: Idiopathic basal ganglia calcification
NBM: Narrative-based medicine
PBC: Primary brain calcification
PDGF: Platelet-derived growth factor
PFBC: Primary familial brain calcification
