# Supplementary figures and images for: Living with primary brain calcification with PDGFB variants: A qualitative study
Source: PLoS One. 2022 Oct 7;17(10):e0275227. doi: 10.1371/journal.pone.0275227 (PMC9543980; doi:10.1371/journal.pone.0275227)

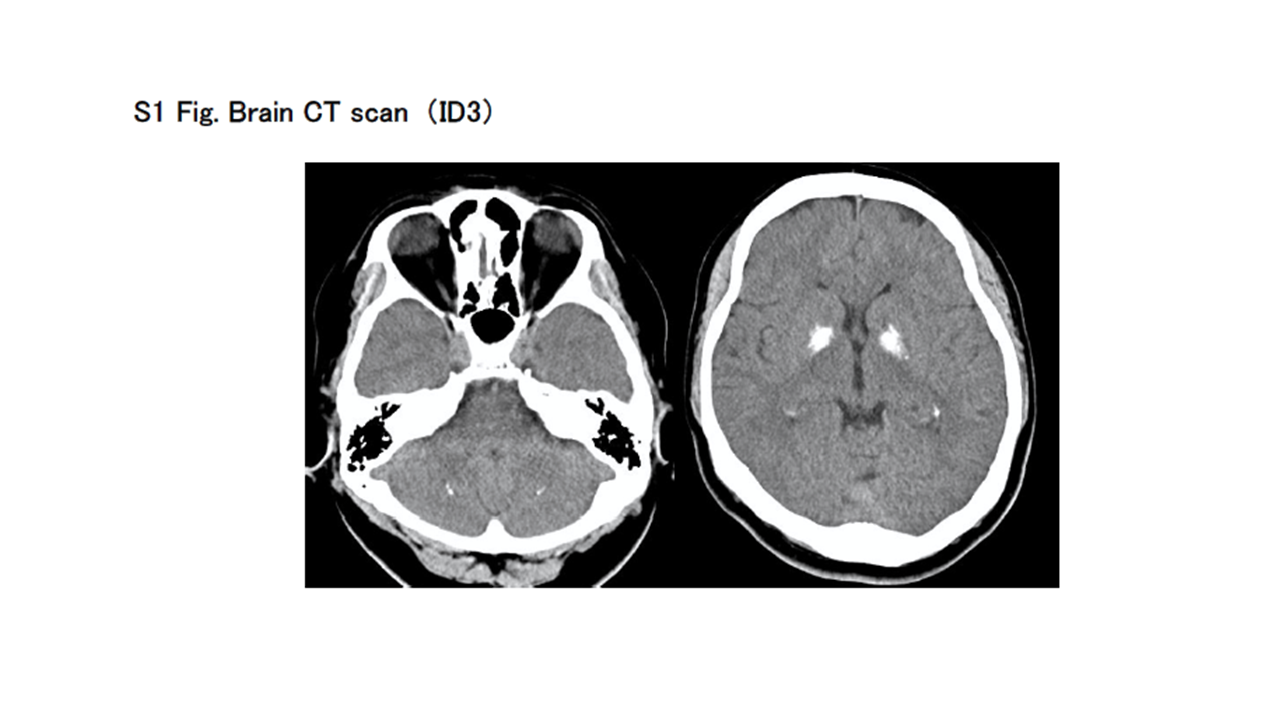

Supplement: S1 Fig — This is the Brain CT scan of ID3. (TIF) [file pone.0275227.s001.tif]

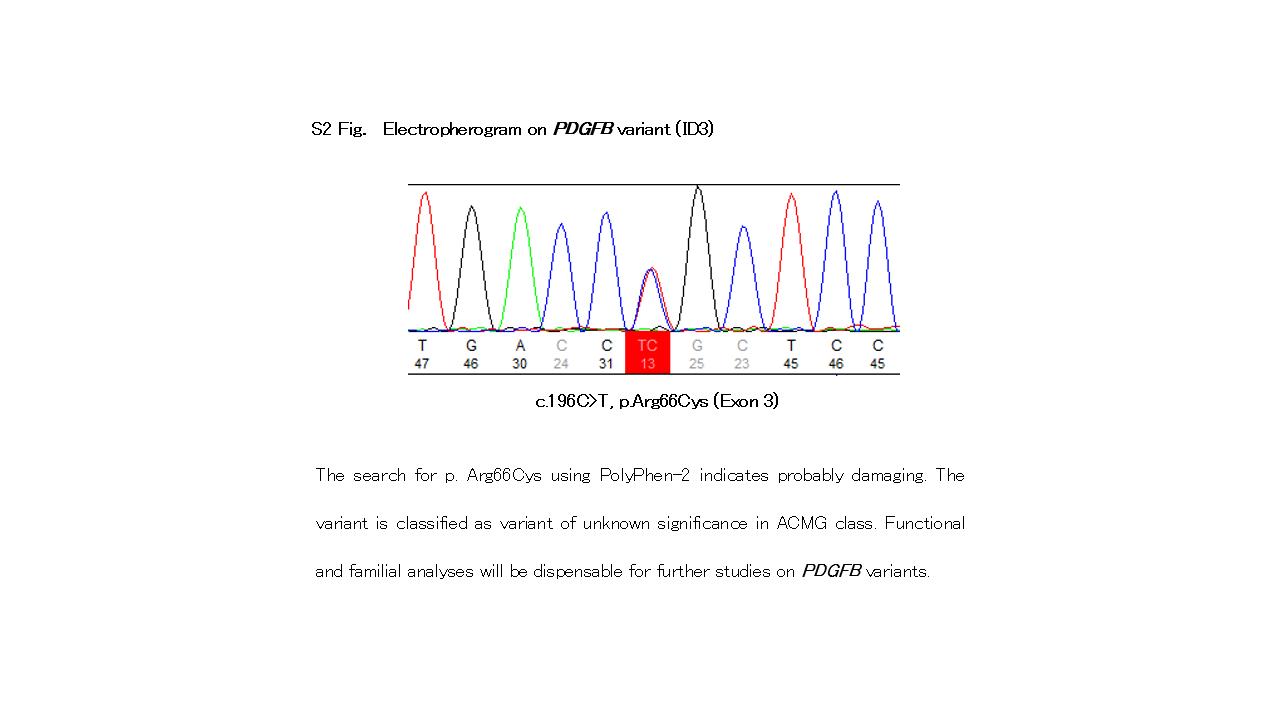

Supplement: S2 Fig — This is the PDGFB Variant of ID3. The search for p. Arg66Cys using PolyPhen-2 indicates probable damage. The variant is classified as a variant of unknown significance in ACMG class. Functional and familial analyses will be dispensable for further studies on PDGFB variants. (TIF) [file pone.0275227.s002.tif]

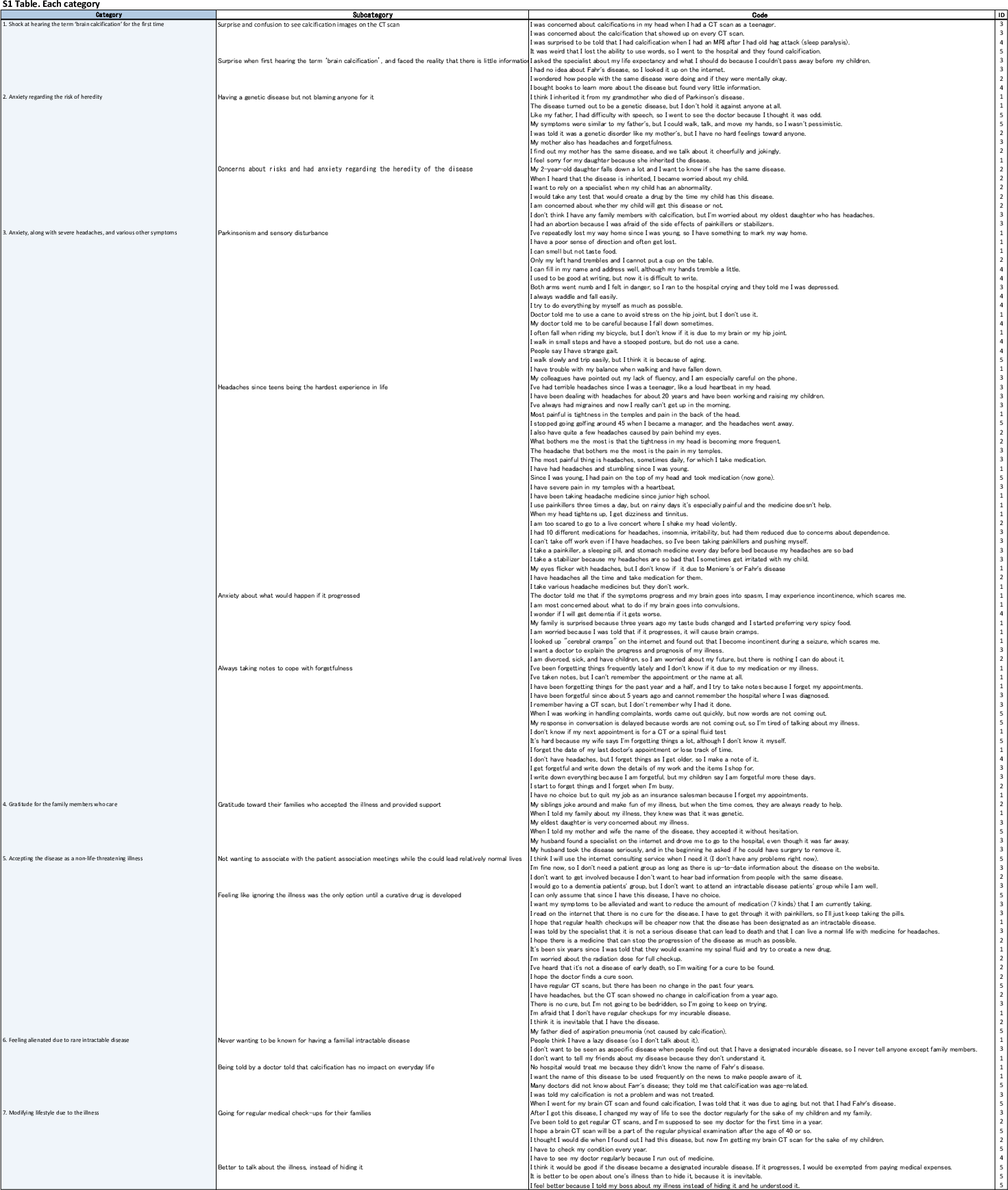

Supplement: S1 Table — This is the complete list of research themes and representative quotes. (TIF) [file pone.0275227.s003.tif]
