# Supplementary material for: Living with primary brain calcification with PDGFB variants: A qualitative study
Source: PLoS One. 2022 Oct 7;17(10):e0275227. doi: 10.1371/journal.pone.0275227 (PMC9543980; doi:10.1371/journal.pone.0275227)
Supplement: S2 Table — This is the entire semi-structured interview. (PDF) [file pone.0275227.s004.pdf]

S2 Table. Interview Guidelines

1. Method

- Each interview is conducted in a private room for approximately one hour.
- If consent to record the interview is obtained, the interview is recorded.

2. Content of the interview

1) Age, family structure, occupation

2) How the disease was discovered
   - What symptoms led you to the hospital?
   - What kind of medical examinations and tests did you undergo?

3) About the disease
   - What kind of explanation did you receive about the disease?
   - How did you feel when you first heard the name of the disease?
   - How did your family react when you told them about your illness?

4) About your worries and anxieties
   - What are you most worried about now?
   - What worries or anxieties have you had in the past?
   - What worries or anxieties do you have about your future life?

5) Are there any new thoughts or ideas that you have gained through the experience of having this illness?

6) About medical care and support systems
   - Do you have any wishes for the medical care system?
   - If there was a patients' group, would you like to join it?

7) Do you have any advice or messages for people with the same disease?

8) Do you have anything you would like to add?
